# Supplementary material for: Tripartite suppression of fission yeast TORC1 signaling by the GATOR1-Sea3 complex, the TSC complex, and Gcn2 kinase
Source: eLife. 2021 Feb 3;10:e60969. doi: 10.7554/eLife.60969 (PMC7857730; doi:10.7554/eLife.60969)
Supplement: Supplementary file 1. [file elife-60969-supp1.docx]

**Supplementary file 1. Fission yeast strains used in this study.**

| **Strains** | **Reference** | **Genotype** |
| --- | --- | --- |
| 972/CA1 | ATCC24843  Laboratory stock | *h-*  Stocked in K. Shiozaki lab. |
| CA101 | Laboratory stock | *h- leu1-32*  Stocked in K. Shiozaki lab. |
| CA8462 | This paper | *h+ seh1:mEGFP(kanMX6)*  Stocked in K. Shiozaki lab. |
| CA8466 | This paper | *h+ seh1Δ::hph*  Stocked in K. Shiozaki lab. |
| CA8509 | This paper | *h- sea3:myc(hph)*  Stocked in K. Shiozaki lab. |
| CA8561 | DOI: 10.7554/eLife.30880 | *h- iml1:myc(hph)*  Stocked in K. Shiozaki lab. |
| CA8594 | DOI: 10.7554/eLife.30880 | *h- P3nmt1:FLAG:npr2(kanMX6)*  Stocked in K. Shiozaki lab. |
| CA8596 | DOI: 10.7554/eLife.30880 | *h- P3nmt1:FLAG:npr3(kanMX6)*  Stocked in K. Shiozaki lab. |
| CA8602 | DOI: 10.7554/eLife.30880 | *h- iml1:myc(hph) P3nmt1:FLAG:npr2(kanMX6)*  Stocked in K. Shiozaki lab. |
| CA8604 | DOI: 10.7554/eLife.30880 | *h- iml1:myc(hph) P3nmt1:FLAG:npr3(kanMX6)*  Stocked in K. Shiozaki lab. |
| CA8606 | This paper | *h- npr2:myc(hph) P3nmt1:FLAG:npr3(kanMX6)*  Stocked in K. Shiozaki lab. |
| CA8659 | This paper | *h- sea2:myc(hph)*  Stocked in K. Shiozaki lab. |
| CA8663 | This paper | *h- sea4:myc(hph)*  Stocked in K. Shiozaki lab. |
| CA8721 | This paper | *h- npr3:myc(hph)*  Stocked in K. Shiozaki lab. |
| CA8749 | This paper | *h- seh1:myc(hph)*  Stocked in K. Shiozaki lab. |
| CA8796 | This paper | *h+ npr2:myc(hph)*  Stocked in K. Shiozaki lab. |
| CA8814 | This paper | *h- seh1Δ::hph sea2Δ::kanMX6 sea4Δ::kanMX6*  Stocked in K. Shiozaki lab. |
| CA8863 | This paper | *h- sea3Δ::hph*  Stocked in K. Shiozaki lab. |
| CA8869 | This paper | *h- seh1Δ::hph sea2Δ::kanMX6 sea3Δ::nat sea4Δ::kanMX6*  Stocked in K. Shiozaki lab. |
| CA8916 | This paper | *h- tsc2Δ::kanMX6*  Stocked in K. Shiozaki lab. |
| CA8922 | This paper | *h+ sec13:FLAG(kanMX6)*  Stocked in K. Shiozaki lab. |
| CA8927 | This paper | *h+ sec13:mEGFP(kanMX6)*  Stocked in K. Shiozaki lab. |
| CA9014 | This paper | *h- iml1:FLAG(kanMX6) sea3:myc(hph)*  Stocked in K. Shiozaki lab. |
| CA9400 | DOI: 10.7554/eLife.30880 | *h- iml1Δ::kanMX6*  Stocked in K. Shiozaki lab. |
| CA9402 | DOI: 10.7554/eLife.30880 | *h- npr2Δ::kanMX6*  Stocked in K. Shiozaki lab. |
| CA9404 | DOI: 10.7554/eLife.30880 | *h- npr3Δ::kanMX6*  Stocked in K. Shiozaki lab. |
| CA9473 | DOI: 10.7554/eLife.30880 | *h- gtr1S16N(kanMX6)*  Stocked in K. Shiozaki lab. |
| CA9494 | This paper | *h- iml1Δ::kanMX6 sea3Δ::hph*  Stocked in K. Shiozaki lab. |
| CA9557 | DOI: 10.7554/eLife.30880 | *h- tsc1Δ::kanMX6*  Stocked in K. Shiozaki lab. |
| CA9560 | DOI: 10.7554/eLife.30880 | *h- gtr1S16N(kanMX6) iml1Δ::hph*  Stocked in K. Shiozaki lab. |
| CA9570 | This paper | *h- tsc1Δ::kanMX6 sea3Δ::hph*  Stocked in K. Shiozaki lab. |
| CA9592 | This paper | *h- gtr1S16N(kanMX6) sea3Δ::hph*  Stocked in K. Shiozaki lab. |
| CA9598 | This paper | *h- npr3Δ::kanMX6 sea3Δ::hph*  Stocked in K. Shiozaki lab. |
| CA9616 | This paper | *h- npr2Δ::kanMX6 sea3Δ::hph*  Stocked in K. Shiozaki lab. |
| CA9901 | This paper | *h- tsc2Δ::kanMX6 sea3Δ::hph*  Stocked in K. Shiozaki lab. |
| CA9907 | DOI: 10.7554/eLife.30880 | *h- iml1Δ::kanMX6 tsc2Δ::hph*  Stocked in K. Shiozaki lab. |
| CA9946 | This paper | *h+ sea3:myc(hph) P3nmt1:FLAG:npr2(kanMX6)*  Stocked in K. Shiozaki lab. |
| CA9948 | This paper | *h- sea3:myc(hph) P3nmt1:FLAG:npr3(kanMX6)*  Stocked in K. Shiozaki lab. |
| CA10082 | This paper | *h- iml1:myc(hph) sea2:FLAG(kanMX6)*  Stocked in K. Shiozaki lab. |
| CA10084 | This paper | *h- iml1:myc(hph) sea3:FLAG(kanMX6)*  Stocked in K. Shiozaki lab. |
| CA10086 | This paper | *h- iml1:myc(hph) sea4:FLAG(kanMX6)*  Stocked in K. Shiozaki lab. |
| CA10132 | This paper | *h- iml1:myc(hph) seh1:FLAG(kanMX6)*  Stocked in K. Shiozaki lab. |
| CA10148 | This paper | *h- iml1:myc(hph) P3nmt1:FLAG:Sec13(kanMX6)*  Stocked in K. Shiozaki lab. |
| CA10178 | This paper | *h- iml1:FLAG(kanMX6) sea2:myc(hph)*  Stocked in K. Shiozaki lab. |
| CA10180 | This paper | *h- iml1:FLAG(kanMX6) sea4:myc(hph)*  Stocked in K. Shiozaki lab. |
| CA11027 | This paper | *h- npr3:myc(hph) P3nmt1:FLAG:npr2(kanMX6)*  Stocked in K. Shiozaki lab. |
| CA11331 | This paper | *h+ sea4:mEGFP:GFP(hph)*  Stocked in K. Shiozaki lab. |
| CA11461 | DOI: 10.7554/eLife.30880 | *h+ iml1:mEGFP:GFP(hph)*  Stocked in K. Shiozaki lab. |
| CA11465 | This paper | *h+ sea2:mEGFP:GFP:mNeonGreen(kanMX6)*  Stocked in K. Shiozaki lab. |
| CA11467 | This paper | *h+ sea3:mEGFP:GFP:mNeonGreen(kanMX6)*  Stocked in K. Shiozaki lab. |
| CA11491 | DOI: 10.7554/eLife.30880 | *h- any1Δ::hph*  Stocked in K. Shiozaki lab. |
| CA11497 | DOI: 10.7554/eLife.30880 | *h- any1Δ::hph iml1Δ::kanMX6*  Stocked in K. Shiozaki lab. |
| CA11751 | DOI: 10.7554/eLife.30880 | *h- iml1:mEGFP:GFP(hph) npr2Δ::kanMX6*  Stocked in K. Shiozaki lab. |
| CA11753 | DOI: 10.7554/eLife.30880 | *h- iml1:mEGFP:GFP(hph) npr3Δ::kanMX6*  Stocked in K. Shiozaki lab. |
| CA12608 | This paper | *h+ P81nmt1:GFP:Npr2(hph)*  Stocked in K. Shiozaki lab. |
| CA12610 | This paper | *h+ P81nmt1:GFP:Npr3(hph)*  Stocked in K. Shiozaki lab. |
| CA12802 | This paper | *h- iml1:myc(hph) P3nmt1:FLAG:npr2(kanMX6) sea3Δ::nat*  Stocked in K. Shiozaki lab. |
| CA12811 | This paper | *h- iml1:myc(hph) P3nmt1:FLAG:npr3(kanMX6) sea3Δ::nat*  Stocked in K. Shiozaki lab. |
| CA12814 | This paper | *h+ P81nmt1:GFP:Npr2(hph) iml1Δ::kanMX6*  Stocked in K. Shiozaki lab. |
| CA12816 | This paper | *h+ P81nmt1:GFP:Npr3(hph) iml1Δ::kanMX6*  Stocked in K. Shiozaki lab. |
| CA12818 | This paper | *h+ P81nmt1:GFP:Npr2(hph) npr3Δ::nat*  Stocked in K. Shiozaki lab. |
| CA12820 | This paper | *h+ P81nmt1:GFP:Npr3(hph) npr2Δ::kanMX6*  Stocked in K. Shiozaki lab. |
| CA13015 | This paper | *h- any1Δ::hph sea3Δ::kanMX6*  Stocked in K. Shiozaki lab. |
| CA14394 | This paper | *h+ iml1:FLAG(kanMX6) sea3:myc(hph) sea4Δ::kanMX6*  Stocked in K. Shiozaki lab. |
| CA14401 | This paper | *h+ iml1:FLAG(kanMX6) sea3:myc(hph) seh1Δ::hph*  Stocked in K. Shiozaki lab. |
| CA14410 | This paper | *h+ iml1:FLAG(kanMX6) sea4:myc(hph) sea3Δ::hph*  Stocked in K. Shiozaki lab. |
| CA14423 | This paper | *h- iml1:FLAG(kanMX6) sea2:myc(hph) sea3Δ::hph*  Stocked in K. Shiozaki lab. |
| CA14442 | This paper | *h+ sea4Δ::nat*  Stocked in K. Shiozaki lab. |
| CA14444 | This paper | *h+ sea2Δ::nat*  Stocked in K. Shiozaki lab. |
| CA14534 | This paper | *h- sec13:FLAG(kanMX6) iml1:myc(hph)*  Stocked in K. Shiozaki lab. |
| CA14606 | This paper | *h- iml1:FLAG(kanMX6) sea3:myc(hph) sea2Δ::nat*  Stocked in K. Shiozaki lab. |
| CA14644 | This paper | *h+ sea2:mEGFP:GFP:mNeonGreen(kanMX6) iml1Δ::kanMX6*  Stocked in K. Shiozaki lab. |
| CA14707 | This paper | *h+ seh1:mEGFP(kanMX6) sea3Δ::hph*  Stocked in K. Shiozaki lab. |
| CA14711 | This paper | *h- sea3:mEGFP:GFP:mNeonGreen(kanMX6) iml1Δ::kanMX6*  Stocked in K. Shiozaki lab. |
| CA14852 | This paper | *h+ sea3:mEGFP:GFP:mNeonGreen(kanMX6) npr3Δ::kanMX6*  Stocked in K. Shiozaki lab. |
| CA14853 | This paper | *h- sea3:mEGFP:GFP:mNeonGreen(kanMX6) npr2Δ::kanMX6*  Stocked in K. Shiozaki lab. |
| CA14866 | This paper | *h- iml1:mEGFP:GFP(hph) sea3Δ::hph*  Stocked in K. Shiozaki lab. |
| CA14868 | This paper | *h- npr2:myc(hph) P3nmt1:FLAG:npr3(kanMX6) sea3Δ::hph*  Stocked in K. Shiozaki lab. |
| CA14869 | This paper | *h+ sea3:myc(hph) P3nmt1:FLAG:npr3(kanMX6) npr2Δ::kanMX6*  Stocked in K. Shiozaki lab. |
| CA14870 | This paper | *h+ sea3:myc(hph) P3nmt1:FLAG:npr2(kanMX6) iml1Δ::kanMX6*  Stocked in K. Shiozaki lab. |
| CA14873 | This paper | *h+ sea3:myc(hph) P3nmt1:FLAG:npr2(kanMX6) npr3Δ::kanMX6*  Stocked in K. Shiozaki lab. |
| CA14934 | This paper | *h- iml1:FLAG(kanMX6) sea3:myc(hph) npr2Δ::kanMX6*  Stocked in K. Shiozaki lab. |
| CA14937 | This paper | *h+ iml1:FLAG(kanMX6) sea3:myc(hph) npr3Δ::kanMX6*  Stocked in K. Shiozaki lab. |
| CA14958 | This paper | *h- sea3:mEGFP:GFP:mNeonGreen(kanMX6) gtr1Q61L(kanMX6)*  Stocked in K. Shiozaki lab. |
| CA14962 | This paper | *h- sea3:mEGFP:GFP:mNeonGreen(kanMX6) gtr1Δ::kanMX6*  Stocked in K. Shiozaki lab. |
| CA14996 | This paper | *h- sea3:myc(hph) P3nmt1:FLAG:npr3(kanMX6) iml1Δ::kanMX6*  Stocked in K. Shiozaki lab. |
| CA15005 | This paper | *h+ sea2:mEGFP:GFP:mNeonGreen(kanMX6) sea3Δ::hph*  Stocked in K. Shiozaki lab. |
| CA15007 | This paper | *h+ sea4:mEGFP:GFP(hph) sea3Δ::hph*  Stocked in K. Shiozaki lab. |
| CA15061 | This paper | *h- sec13:FLAG(kanMX6) iml1:myc(hph) sea3Δ::hph*  Stocked in K. Shiozaki lab. |
| CA15091 | This paper | *h- iml1:myc(hph) sea3Δ::hph*  Stocked in K. Shiozaki lab. |
| CA15092 | This paper | *h+ npr2:myc(hph) sea3Δ::hph*  Stocked in K. Shiozaki lab. |
| CA15093 | This paper | *h- npr3:myc(hph) sea3Δ::hph*  Stocked in K. Shiozaki lab. |
| CA15262 | This paper | *h- iml1:FLAG(kanMX6) seh1:myc(hph)*  Stocked in K. Shiozaki lab. |
| CA15271 | This paper | *h+ iml1:FLAG(kanMX6) seh1:myc(hph) sea3Δ::hph*  Stocked in K. Shiozaki lab. |
| CA16273 | This paper | *h- P81nmt1:GFP:npr2(hph) sea3Δ::hph*  Stocked in K. Shiozaki lab. |
| CA16277 | This paper | *h+ P81nmt1:GFP:Npr3(hph) sea3Δ::hph*  Stocked in K. Shiozaki lab. |
| CA6425 | This paper | *h90*  Stocked in K. Shiozaki lab. |
| CA10128 | This paper | *h90 iml1Δ::hph*  Stocked in K. Shiozaki lab. |
| CA10951 | This paper | *h90 tsc2Δ::kanMX6*  Stocked in K. Shiozaki lab. |
| CA13699 | This paper | *h- iml1:myc(hph) gtr1:FLAG(kanMX6)*  Stocked in K. Shiozaki lab. |
| CA16708 | This paper | *h- iml1:myc(hph) gtr1:FLAG(kanMX6) sea3Δ::hph*  Stocked in K. Shiozaki lab. |
| CH59 | NBPR FY7551 (https://yeast.nig.ac.jp/yeast/) | *h+ arg6*  Stocked in T. Kanki lab. |
| TFSP1055 | This paper | *h- leu1-32 pgk1:mEGFP(kanMX6) P3nmt1:FLAG:yih1(hph)*  Stocked in T. Kanki lab. |
| TFSP1143 | This paper | *h- gcn2Δ::hph iml1Δ::nat*  Stocked in T. Kanki lab. |
| TFSP1145 | This paper | *h- gcn2Δ::hph tsc2Δ::nat*  Stocked in T. Kanki lab. |
| TFSP1753 | This paper | *h- sea3Δ::kanMX6*  Stocked in T. Kanki lab. |
| TFSP1755 | This paper | *h- sea3Δ::kanMX6 gcn2Δ::hph*  Stocked in T. Kanki lab. |
| TFSP1983 | This paper | *h- pgk1:mEGFP(kanMX6) gcn2Δ::ble*  Stocked in T. Kanki lab. |
| TFSP1985 | This paper | *h- pgk1:mEGFP(kanMX6) iml1Δ::nat*  Stocked in T. Kanki lab. |
| TFSP1987 | This paper | *h- pgk1:mEGFP(kanMX6) tsc2Δ::nat*  Stocked in T. Kanki lab. |
| TFSP1989 | This paper | *h- pgk1:mEGFP(kanMX6) gcn2Δ::hph iml1Δ::nat*  Stocked in T. Kanki lab. |
| TFSP1991 | This paper | *h- pgk1:mEGFP(kanMX6) gcn2Δ::hph tsc2Δ::nat*  Stocked in T. Kanki lab. |
| TFSP2053 | This paper | *h- pgk1:mEGFP(kanMX6) tsc2Δ::nat iml1Δ::hph*  Stocked in T. Kanki lab. |
| TFSP2535 | This paper | *h- tsc1Δ::kanMX6 gcn2Δ::hph*  Stocked in T. Kanki lab. |
| TFSP2611 | This paper | *h- pgk1:mEGFP(kanMX6) gcn2Δ::ble tsc2Δ::nat iml1Δ::hph*  Stocked in T. Kanki lab. |
| TFSP2671 | This paper | *h- pgk1:mEGFP(kanMX6) fil1Δ::nat*  Stocked in T. Kanki lab. |
| TFSP2829 | This paper | *h- leu1-32 fil1Δ::nat*  Stocked in T. Kanki lab. |
| TFSP2831 | This paper | *h- pgk1:mEGFP(kanMX6) fil1Δ::nat*  Stocked in T. Kanki lab. |
| TFSP287 | This paper | *h- gcn2Δ::hph*  Stocked in T. Kanki lab. |
| TFSP2871 | This paper | *h- fil1Δ::nat*  Stocked in T. Kanki lab. |
| TFSP289 | This paper | *h- leu1-32 gcn2Δ::hph*  Stocked in T. Kanki lab. |
| TFSP2921 | This paper | *h- leu1-32 fil1:FLAG(hph) gcn2Δ::nat*  Stocked in T. Kanki lab. |
| TFSP2923 | This paper | *h- leu1-32 ura4-D18 tif211S52A(ura4) fil1:FLAG(hph)*  Stocked in T. Kanki lab. |
| TFSP2925 | This paper | *h- leu1-32 fil1:FLAG(hph)*  Stocked in T. Kanki lab. |
| TFSP3055 | This paper | *h- fil1Δ::nat sea3Δ::kan*  Stocked in T. Kanki lab. |
| TFSP3085 | This paper | *h- fil1Δ::nat tsc2Δ::kanMX6*  Stocked in T. Kanki lab. |
| TFSP3109 | This paper | *h+ arg6 pgk1:mEGFP(kanMX6) gcn2Δ::nat*  Stocked in T. Kanki lab. |
| TFSP3137 | This paper | *h- gcn2Δ::ble tsc2Δ::kan iml1Δ::nat*  Stocked in T. Kanki lab. |
| TFSP731 | This paper | *h- leu1-32 pgk1:mEGFP(kanMX6)*  Stocked in T. Kanki lab. |
| TFSP737 | This paper | *h- pgk1:mEGFP(kanMX6) gcn2Δ::hph*  Stocked in T. Kanki lab. |
| TFSP739 | This paper | *h- leu1-32 pgk1:mEGFP(kanMX6) gcn2Δ::hph*  Stocked in T. Kanki lab. |
| TFSP763 | This paper | *h- pgk1:mEGFP(kanMX6) gcn3Δ::hph*  Stocked in T. Kanki lab. |
| TFSP771 | This paper | *h- pgk1:mEGFP(kanMX6) gcn1Δ::hph*  Stocked in T. Kanki lab. |
| TFSP779 | This paper | *h- pgk1:mEGFP(kanMX6) gcn20Δ::hph*  Stocked in T. Kanki lab. |
| TFSP781 | This paper | *h- pgk1:mEGFP(kanMX6) cpc2Δ::nat*  Stocked in T. Kanki lab. |
| TFSP787 | This paper | *h- pgk1:mEGFP(kanMX6) isp6Δ::nat*  Stocked in T. Kanki lab. |
| TFSP825 | This paper | *h- ura4-D18 tif211S52A(ura4)*  Stocked in T. Kanki lab. |
| TFSP827 | This paper | *h- leu1-32 ura4-D18 tif211S52A(ura4)*  Stocked in T. Kanki lab. |
| TFSP829 | This paper | *h- leu1-32 pgk1:mEGFP(kanMX6) iml1Δ::hph*  Stocked in T. Kanki lab. |
| TFSP837 | This paper | *h+ arg6 pgk1:mEGFP(kanMX6)*  Stocked in T. Kanki lab. |
| TFSP855 | This paper | *h- leu1-32 ura4-D18 tif211S52A(ura4) pgk1:mEGFP(kanMX6)*  Stocked in T. Kanki lab. |
| TFSP919 | This paper | *h- leu1-32 pgk1:mEGFP(kanMX6) tsc2Δ::nat*  Stocked in T. Kanki lab. |
| TFSP925 | This paper | *h- ura4-D18 tif211S52A(ura4) pgk1-mEGFP:kan*  Stocked in T. Kanki lab. |
| TFSP945 | This paper | *h- pgk1:mEGFP(kanMX6) atg7Δ::hph*  Stocked in T. Kanki lab. |
| TFSP95 | This paper | *h- pgk1:mEGFP(kanMX6)*  Stocked in T. Kanki lab. |
| TFSP967 | This paper | *h- leu1-32 pgk1:mEGFP(kanMX6) iml1Δ::hph tsc2Δ::nat*  Stocked in T. Kanki lab. |
| TFSP4321 | This paper | *h- fil1-FLAG::nat iml1Δ::kanMX6 tsc2Δ::hph*  Stocked in T. Kanki lab. |
| TFSP4333 | This paper | *h- gcn2Δ::ble tsc2Δ::kanMX6 sea3Δ::hph*  Stocked in T. Kanki lab. |
| TFSP4335 | This paper | *h- gcn2Δ::ble tsc2Δ::kanMX6 sea3Δ::hph any1Δ::nat*  Stocked in T. Kanki lab. |
| TFSP4343 | This paper | *h90 gcn2Δ::nat*  Stocked in T. Kanki lab. |
| TFSP4317 | This paper | *h90 iml1Δ::hph gcn2Δ::nat*  Stocked in T. Kanki lab. |
| TFSP4331 | This paper | *h90 iml1Δ::hph tsc2Δ::kanMX6*  Stocked in T. Kanki lab. |
| TFSP4319 | This paper | *h90 tsc2Δ::kanMX6 gcn2Δ::nat*  Stocked in T. Kanki lab. |
| TFSP4347 | This paper | *h90 gcn2Δ::nat tsc2Δ::kanMX6 iml1Δ::hph*  Stocked in T. Kanki lab. |
| TFSP753 | This paper | *h- leu1-32 pgk1:mEGFP(kanMX6) hri1Δ::nat*  Stocked in T. Kanki lab. |
| TFSP751 | This paper | *h- leu1-32 pgk1:mEGFP(kanMX6) hri2Δ::nat*  Stocked in T. Kanki lab. |
| TFSP755 | This paper | *h- leu1-32 pgk1:mEGFP(kanMX6) hri1Δ::hph hri2Δ::nat*  Stocked in T. Kanki lab. |
